# Decoding the complete arsenal for cellulose and hemicellulose deconstruction in the highly efficient cellulose decomposer *Paenibacillus* O199

**DOI:** 10.1186/s13068-016-0518-x

**Published:** 2016-05-14

**Authors:** Rubén López-Mondéjar, Daniela Zühlke, Tomáš Větrovský, Dörte Becher, Katharina Riedel, Petr Baldrian

**Affiliations:** Laboratory of Environmental Microbiology, Institute of Microbiology of the CAS, v. v. i., Průmyslová 595, 252 42 Vestec, Czech Republic; Institute of Microbiology, Ernst-Moritz-Arndt-University of Greifswald, Friedrich-Ludwig-Jahnstrasse 15, 17487 Greifswald, Germany

**Keywords:** Cellulose, Hemicellulose, *Paenibacillus*, Glycosyl hydrolase, CAZyme, Plant biomass

## Abstract

**Background:**

The search for new enzymes and microbial strains to degrade plant biomass is one of the most important strategies for improving the conversion processes in the production of environment-friendly chemicals and biofuels. In this study, we report a new *Paenibacillus* isolate, O199, which showed the highest efficiency for cellulose deconstruction in a screen of environmental isolates. Here, we provide a detailed description of the complex multi-component O199 enzymatic system involved in the degradation of lignocellulose.

**Results:**

We examined the genome and the proteome of O199 grown on complex lignocellulose (wheat straw) and on microcrystalline cellulose. The genome contained 476 genes with domains assigned to carbohydrate-active enzyme (CAZyme) families, including 100 genes coding for glycosyl hydrolases (GHs) putatively involved in cellulose and hemicellulose degradation. Moreover, 31 % of these CAZymes were expressed on cellulose and 29 % on wheat straw. Proteomic analyses also revealed a complex and complete set of enzymes for deconstruction of cellulose (at least 22 proteins, including 4 endocellulases, 2 exocellulases, 2 cellobiohydrolases and 2 β-glucosidases) and hemicellulose (at least 28 proteins, including 5 endoxylanases, 1 β-xylosidase, 2 xyloglucanases, 2 endomannanases, 2 licheninases and 1 endo-β-1,3(4)-glucanase). Most of these proteins were secreted extracellularly and had numerous carbohydrate-binding domains (CBMs). In addition, O199 also secreted a high number of substrate-binding proteins (SBPs), including at least 42 proteins binding carbohydrates. Interestingly, both plant lignocellulose and crystalline cellulose triggered the production of a wide array of hydrolytic proteins, including cellulases, hemicellulases, and other GHs.

**Conclusions:**

Our data provide an in-depth analysis of the complex and complete set of enzymes and accessory non-catalytic proteins—GHs, CBMs, transporters, and SBPs—implicated in the high cellulolytic capacity shown by this bacterial strain. The large diversity of hydrolytic enzymes and the extracellular secretion of most of them supports the use of *Paenibacillus* O199 as a candidate for second-generation technologies using paper or lignocellulosic agricultural wastes.

**Electronic supplementary material:**

The online version of this article (doi:10.1186/s13068-016-0518-x) contains supplementary material, which is available to authorized users.

## Background

Concerns about the non-renewable nature of fossil fuels and their rapid consumption together with their effects on the global climate have driven the search for alternative sources of energy [1]. In this context, plant biomass represents a renewable and abundant source for the production of environmentally friendly chemicals and biofuels [2]. Plant biomass is composed of lignocellulose, a highly organized and interlinked mix of different polymers containing mainly cellulose (35–50 %), hemicelluloses (20–50 %) and lignin (10–35 %) [1]. Plant biomass represents a recalcitrant structure whose decomposition is limited by several factors, such as (1) the complex structure of lignin, (2) the crystalline organization of cellulose fibrer and (3) the diversity of hemicellulases; its degradation into simple compounds, such as monosaccharides, may represent a challenging process [3, 4]. Although the chemical hydrolysis of lignocellulosic biomass may theoretically overcome these limitations, some secondary products formed in this process may inhibit fermentation, which represents the following step in the production of biofuels. The use of microbial enzymes and enzymatic hydrolysis can overcome these issues and represents a ‘greener’ technology [2, 5]. Cellulases are the most important enzymes in this process, cleaving the β-1,4 bond in the cellulose chain and are traditionally classified as endocellulases (cleavage inside the cellulose chain), exocellulases or cellobiohydrolases (acting on the ends of the chain) and β-glucosidases (converting cellobiose to glucose monomers). Most enzymes with cellulolytic activity belong to one of the above groups of hydrolases (glycoside hydrolases, GHs), a subgroup of the carbohydrate-active enzymes (CAZymes) [6]. Enzymes with cellulolytic activity are mainly found in the families GH1, GH3, GH5, GH6, GH7, GH8 GH9, GH12, GH45, and GH48. Similar to cellulases, hemicellulases cleave the various bonds within hemicellulose and are classified based on their mode of action and substrate preference into endoxylanases, xylosidases, xyloglucanases, endomannanases, mannosidases, fucosidases, arabinofuranosidases, and others. Hemicellulolytic enzymes are principally found in families GH2, GH10, GH11, GH16, GH26, GH30, GH31, GH36, GH43, GH51, GH74 and GH95. Importantly, members of the same GH family may catalyse different reactions, and their family membership may not sufficiently indicate the targets of their activity. Therefore, the comparison of protein sequences with biochemically characterized CAZymes is essential for an accurate classification [7]. Together with GHs, other CAZymes may also be involved in the degradation of cellulose and hemicellulose, such as xylan esterases (CE), lyases (PL) and lytic polysaccharide monooxygenases (LPMO) from families AA9 and AA10 [8]. Moreover, both cellulases and hemicellulases may contain carbohydrate-binding modules (CBM) that bind cellulose or hemicelluloses and are essential for effective hydrolysis [1].

The exploration of environments where lignocellulose represents an important resource is a promising strategy for discovering new microorganisms and enzymes useful for biomass conversion and biofuel production [9]. In these natural habitats, plant biomass is mainly decomposed by fungi, bacteria and protozoa. Several fungi are well known as powerful producers of cellulolytic and hemicellulolytic enzymes for plant biomass conversion to sugars [10]; however, studies exploring diverse and versatile bacteria have led to the discovery of novel cellulases, some of which exhibit better properties than fungal ones and can thus reduce the economic costs of the conversion process [11]. In this context, the biomass-rich soils from forests are considered a “gold mine” for identifying new bacterial strains and novel enzymatic systems, which are extremely resistant to environmental stresses occurring in these ecosystems and may be able to survive the harsh conditions in biomass conversion and biofuel production [12, 13]. Several strains have been isolated from soil, and numerous cellulases and hemicellulases have been individually studied and characterized [2].

The genus *Paenibacillus* belonging to *Firmicutes* is known to include strains able to produce enzymes for industrial and agricultural applications, and numerous strains have recently been described as cellulolytic or hemicellulolytic [5, 14, 15]. In addition, cellulases and xylanases have been purified and described from the members of this genus [5, 16, 17]. However, the whole enzymatic complement of *Paenibacillus* spp. has not been systematically explored, although this is necessary for a complete understanding of the biodegradative potential of this genus, considering the synergistic mode of action of the enzymes. Although some recent works were focused on the study of multienzyme complexes [18, 19], the studies limited to the analysis of catalytic efficiency of individual enzymes and their combinations are insufficient for the complete evaluation of the potential of *Paenibacillus* spp. in the degradation of lignocellulose [14].

New molecular methods are useful for exploring the potential of bacterial strains as plant biomass decomposers [20]. The sequencing and analysis of bacterial genomes have revealed differences in the potential mechanisms of cellulose degradation and have been used repeatedly for the prediction of the cellulose and hemicellulose degradation potential of bacterial taxa based on the presence of specific CAZyme families [21]. However, the presence of cellulose- and hemicellulase-encoding genes in a genome does not necessarily imply that the bacterial strain is able to degrade plant biomass [22], and proteomic approaches on cellulose-grown cells are therefore required to provide the link between the genomic potential and the actual expression [23, 24].

The aim of this study was to explore the cellulolytic and hemicellulolytic abilities of the bacterium *Paenibacillus* sp. O199. This novel strain was isolated from forest soil, where it exhibited the highest efficiency of cellulose deconstruction among the screened isolates. Whole-genome sequencing and annotation were combined with GeLC-MS/MS to characterize its extracellular proteome during growth on plant biomass and on microcrystalline cellulose. Our results reveal the presence of a complex multi-component enzymatic system that is expressed during the degradation of cellulose and complex lignocellulose, indicating that this strain may have a high potential for biotechnology applications.

## Results

### Identification and cellulolytic activity of the *Paenibacillus* O199 strain

The bacterial isolate O199 degraded carboxymethylcellulose (CMC) during incubation on agar plates. More importantly, it was highly efficient at degrading cellulosic filter paper during growth in liquid media, degrading it in less than one week, which was faster than any other isolate screened from forest soil (Additional file 1: Figure S1A). The measurement of enzymatic activities after incubation showed the production of several enzymes involved in the deconstruction of plant polysaccharides (Additional file 1: Figure S1B).

The comparison of the 16S rRNA gene of the strain O199 with the reference 16S rRNA gene sequences of the type strains in the EzTaxon server showed the closest matches to *P. tundrae* (with a pairwise similarity of 99.86 %), *P. tylopili* (99.65 %), *P. xylanexedens* (98.65 %), and *P. amylolyticus* (99.43 %), all of which also clustered together in the phylogenetic tree (Additional file 2: Figure S2). The isolate was named *Paenibacillus* sp. O199.

### Genomic features of *Paenibacillus* O199

**Fig. 1:**
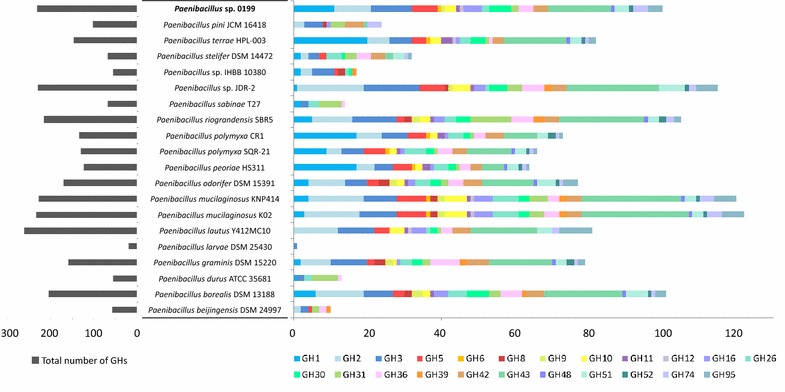
Predicted numbers of glycosyl hydrolases (GH) in the genome of *Paenibacillus* O199 and the genomes of other species of *Paenibacillus*. On the *left* total number of GHs found in the genome; on the *right* gene content in GH families containing enzymes involved in the degradation of cellulose and hemicelluloses

The draft genome assembly indicated a genome size of 7,193,447 bases. Annotation predicted 6507 protein-coding sequences, including 72 RNA genes and 453 predicted SEED subsystem features. Among the predicted proteins, 476 (7.3 %) had one or more domains assigned to CAZyme families, including 231 GHs, 82 CBMs, 10 AAs, 79 CEs, and 13 PLs (Additional file 3: Table S1). The putative genes encoding GHs belonged to 61 different families with gene counts ranging between 1 and 28. The total content of GHs was among the highest so far recorded in the genomes of *Paenibacillus* species (Fig. 1).

Numerous genes assigned to GH families involved in cellulose and hemicellulose deconstruction were detected. For example, genes belonging to the cellulolytic families GH6, GH9, GH12 or GH48 were found in single copies in the genome, and genes from families GH1, GH2, GH3, GH5, GH16, GH30 and GH43 encoding putative β-glucosidases, β-xylanases and other hemicellulases were more abundant. The number of genes belonging to these families was high (100) in comparison with most of the sequenced strains of *Paenibacillus* but similar to those strains isolated from soil and the rhizosphere, such as *P. borealis* (101), *P. riograndensis* (105) or *P. terrae* (82) (Fig. 1). Furthermore, the genome was also rich in proteins with CBM domains involved in cellulose and hemicellulose binding, such as CBM3, CBM6, CBM9, CBM35 and CBM46. Multiple carbohydrate esterases (CE) were also detected in the genome, including families encoding enzymes that act on xylan, such as CE1, CE3, CE4, and CE7, among others. A gene encoding a lytic polysaccharide monooxygenase from the family AA10 was also identified.

In general, genes encoding potential cellulases and hemicellulases were randomly distributed in the genome, with the exception of some genes organized in short clusters. CAZymes were flanked by numerous genes coding for proteins involved in nutrient transport and by transcriptional regulators. The main transporters were annotated as ATP-binding cassette (ABC transporters) systems and phosphotransferase systems. In addition, numerous genes encoding transcriptional regulators (such as AraC, TetR, DeoR, LysR, and GntR, among others), two-component regulatory systems, and sigma factors were also frequently found flanking the CAZymes.

### Expression of proteins during growth on crystalline cellulose and wheat straw

Analysis by GeLC-MS/MS allowed us to identify 961 proteins in the extracellular fraction of *Paenibacillus* O199 (Additional file 4: File SF1). Approximately 68 % of the proteins were expressed in both cellulose- and wheat straw-supplemented cultures, whereas 228 proteins (23.7 %) were exclusively expressed in cellulose, and 77 (around the 8 %) were found only with wheat straw (Additional file 5: Figure S3A). A higher number of expressed proteins showed no functional prediction or were not classified in any functional categories (Additional file 5: Figure S3B). However, many proteins involved in energy metabolism and in transport and binding of nutrients were found in the exoproteome. Even if some of these proteins showed higher abundance during growth on cellulose or on straw (e.g., in the case of proteins involved in sugar metabolism or in transport of carbohydrates, organic alcohols and acids), most proteins showed similar expression in both carbon sources (Additional file 5: Figure S3C).

### Carbon source-dependent expression of carbohydrate-active enzymes

**Fig. 2:**
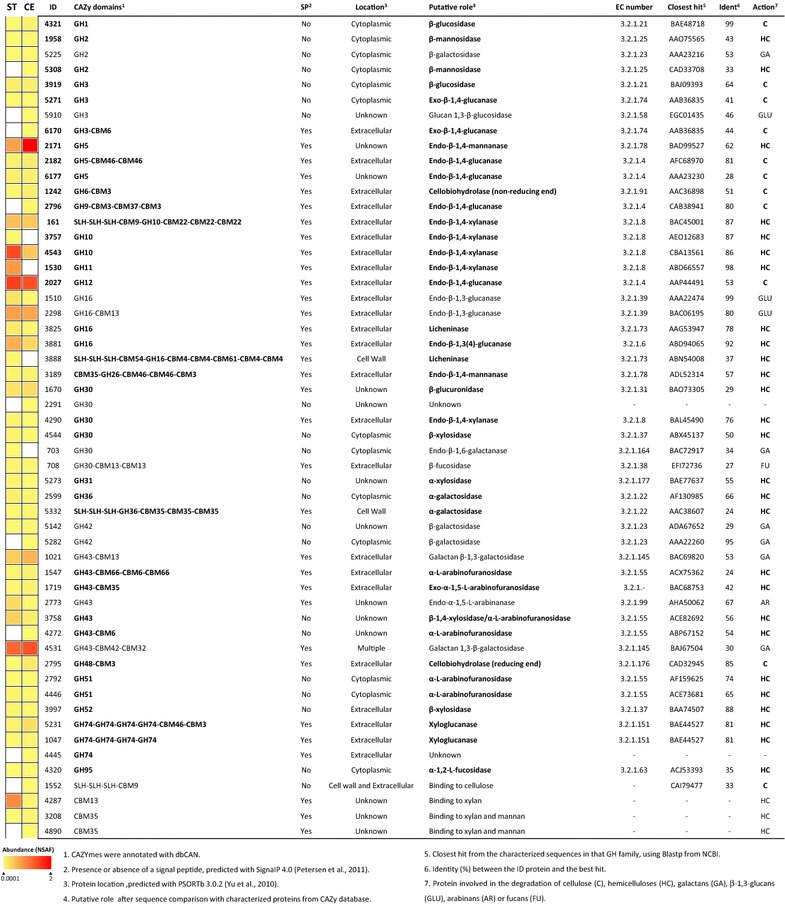
Expression of *Paenibacillus* O199 proteins involved in cellulose and hemicellulose degradation during the growth on wheat straw (ST) and cellulose (CE). Protein abundance is colour-coded, increasing from *yellow* to *red*, and CAZymes not found are in *white*. Proteins with putative role as cellulases and hemicellulases are highlighted in *bold*

**Fig. 3:**
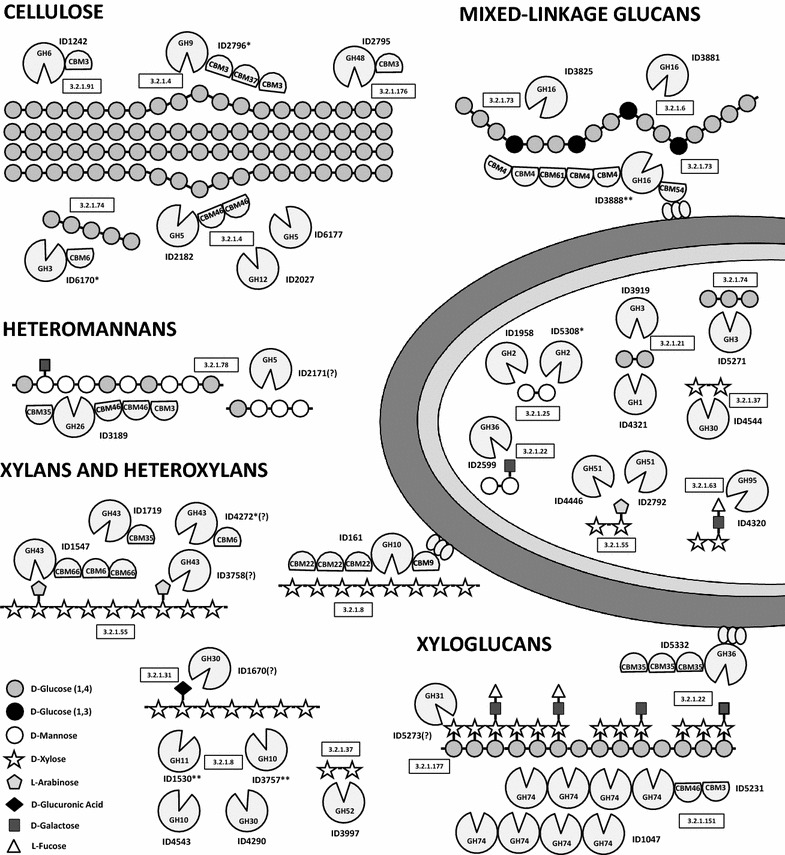
Simplified schematic overview of all the proteins expressed by *Paenibacillus* O199, their role in the hydrolysis of cellulose and hemicellulose (xylan, glucomannan, xyloglucan and mixed-linkage glucans), and their location. Only proteins characterized as cellulases and hemicellulases are shown. Proteins marked with an *asterisk* were only expressed on cellulose, and proteins marked with a *double asterisk* were only expressed on wheat straw. *Question marks* indicate unclear location. Polysaccharides structures are based on Burton et al. [65]

The percentages of expressed CAZymes were slightly higher on cultures growing on crystalline cellulose (31 %) than on wheat straw (29 %), with the exception of CEs and PLs, which showed higher diversity on straw. Twenty-two GH families containing proteins involved in the degradation of cellulose and hemicellulose were detected in the proteome of *Paenibacillus* O199 (Fig. 2, Additional file 4: File SF1). The strain expressed four endoglucanases, two exocellulases, two β-glucosidases and two cellobiohydrolases, although one of the endocellulases (ID2796) and one of the exocellulases (ID6170) were exclusively found on cellulose. *Paenibacillus* O199 also produced at least twenty-eight proteins degrading xylan (five endoxylanases and one β-xylosidase), xyloglucan (including two xyloglucanases), glucomannan (two endomannanases) and mixed-linkage glucans (two licheninases and one endo-β-1,3(4)-glucanase), which acted on numerous residues of hemicellulose chains, and three of the proteins were only found in straw. Many proteins showed multiple domains, including one or more CBMs. The endoxylanase ID161 and the endomannanase ID3189 each contained four different domains, and the licheninase ID3888 contained six. ID3888, ID161 and the α-galactosidase ID5332 also contained three copies of a surface-layer homology (SLH) motif for cell wall anchoring. Most of the proteins degrading cellulose and hemicelluloses were predicted to be extracellularly located, with the exception of enzymes hydrolysing disaccharides and other oligosaccharides (β-glucosidases, β-xylanase or β-mannanases), which had cytoplasmic locations (Fig. 2). The summary of the expressed proteins involved in cellulose and hemicellulose degradation and their different targets within plant biopolymers is schematically shown in Fig. 3.

Other CAZymes involved in the cleavage of arabinans (GH43), galactans (GH2, GH30, GH42, GH43 and GH53) and rhamnogalacturonans (GH28 and GH105) from pectin chains were also recorded (Additional file 4: File SF1). Additionally, enzymes involved in chitin or peptidoglycan (GH18) and other glucan degradation (GH13, GH16) and GHs involved in general bacterial metabolism, such as glycan biosynthesis and catabolism (*N*-acetylgalactosaminidases) (GH109), were highly expressed. Interestingly, some proteins from GH and CBM families not involved in cellulose degradation, such as GH129, GH130 and CBM35, were exclusively found during growth on crystalline cellulose. Moreover, proteins from families CE1, CE3, CE7 and CE12, containing putative acetyl xylan esterases, and from families PL1, PL3, PL4, PL9, PL11, involved in pectin degradation, were expressed not only on straw but also on cellulose, where their substrates were not present. Lastly, an LMPO from the AA10 containing a CBM12 domain and putatively involved in chitin degradation was also produced on both carbon sources (Additional file 4: File SF1).

### Carbon source-dependent expression of other proteins involved in binding and uptake of nutrients

Interestingly, numerous proteins involved in the transport and binding of nutrients were found in the exoproteome of *Paenibacillus* O199 under both analysed conditions (Additional file 5: Figure S3B and C). Most of them belonged to the subunit substrate-binding protein (SBP) from the ABC transporters, encompassing approximately 9.5 % of the total detected proteins. SBPs have been defined as key determinants for the specificity and affinity of ABC uptake systems in bacteria [25]. In our study, almost ninety different SBPs binding multiple monosaccharides (such as xylose, galactose or rhamnose), polysaccharides, oligopeptides, vitamins and microelements were produced in the bacterial cultures. Among them, at least forty-two proteins were involved in the binding of carbohydrates and fourteen in the binding of amino acids and peptides (Additional file 6: Table S2). Ten of them were specific for growth on straw and twelve for growth on cellulose (Additional file 4: File SF1).

## Discussion

The search for new bacterial strains and enzymes to efficiently degrade plant biomass represents one of the most promising approaches to optimize biofuel production [26]. In this study, a new strain isolated from forest soil showed high cellulolytic efficiency, being able to degrade filter paper strips in 7 days, which was much faster than any other cellulolytic isolate tested. The isolate was identified as a member of the genus *Paenibacillus*. Several strains of this globally abundant genus of soil bacteria have been already described as cellulolytic or hemicellulolytic (Figure S2). Due to the promising characteristics of these strains in biomass conversion, several genomes from cellulolytic and hemicellulolytic strains of *Paenibacillus* have recently been published [27–30]. However, no in-depth analyses have been carried out on them so it is unclear to what extent their genomes are expressed. Here, the genome of *Paenibacillus* O199 was sequenced, assembled and analysed to obtain complete information about the potential polysaccharide hydrolysis. The published genomes of four strains of *Paenibacillus polymyxa* were analysed and compared recently [31], not only focusing mainly on the genes related to plant growth promotion but also noting a large arsenal of hydrolytic enzymes for plant biomass degradation. The genome of *Paenibacillus* O199 contained numerous genes encoding CAZymes putatively involved in plant polymer degradation, which together represented a higher percentage of its genome than other well-known cellulolytic bacteria, such as *Clostridium thermocellum*, *C. japonicus* or *Streptomyces* sp. ActE [24]. The correlation between a high number of CAZymes in the genome and plant biomass degradation has been suggested by several authors [32, 33]. Moreover, the number of genes belonging to GH families involved in cellulose and hemicellulose degradation in O199 was comparable to other described plant biomass-degrading strains, such as *Paenibacillus* sp. JDR-2 (Fig. 1). In particular, some of these specific GH families (especially GH5, GH6, GH9, GH12 and GH48) have been identified as the main mediators of cellulose degradation by Gram-positive bacteria [7]. Furthermore, a large number of other GHs involved in the degradation of different biopolymers, such as chitin, peptidoglycan, starch or pectin, were found in the genome of *Paenibacillus* O199. The presence of diverse gene sets involved in the degradation of various biopolymers has also been reported from the genomes of other *Paenibacillus* strains isolated from soil [29, 31].

Unfortunately, genome analyses only indicate the functional potential of bacteria and not their real activity [22, 31]. Here, we have demonstrated that the potential cellulases and hemicellulases encoded in the genome of *Paenibacillus* O199 were produced on crystalline cellulose and on a complex lignocellulosic substrate, but the expressed proteins represented only a small fraction (approximately 30 %) of those predicted by genome sequencing. Similar results have been described in other cellulolytic bacteria, where only a fraction of predicted CAZymes were expressed [24]. Despite the absence of some predicted CAZymes, the expressed proteins in O199 still represented a complete system for cellulose deconstruction, with multiple endoglucanases, β-glucosidases, exoglucanases and cellobiohydrolases (Figs. 2, 3), responsible for the powerful activity shown by this strain. Interestingly, enzymes such as endoglucanases, exoglucanases and β-glucosidases were redundant, showing different variants of secreted enzymes. The redundancy of hydrolytic enzymes has been typically explained by synergistic effects observed during biopolymer degradation by enzyme mixtures and has been found in many other bacterial strains [15, 34]. For example, Gastelum-Arellanez et al. [14] observed that the endoglucanase activity of a cellulolytic strain of *P. polymyxa* was due to at least fourteen different expressed proteins. Similar results were found in *Paenibacillus* O199, which expressed at least four different endoglucanases, one of which (ID2796) exhibited the typical characteristics of processive endoglucanases [35]: the GH9 and CBM3 domains. Moreover, this enzyme seemed to act synergistically with a GH48 cellobiohydrolase (ID2795) encoded by a contiguous gene for degrading crystalline cellulose. Similar structures with contiguous genes encoding GH9 and GH48 cellulases have been reported in other strains of *Paenibacillus* sp. and in *C. thermocellum* [36, 37]. Redundancy was also found in proteins involved in hemicellulose degradation, such as endoxylanases, xyloglucanases, mannanases and licheninases, which also displayed diverse structures with GH domains from different families within a single protein and with multiple CBMs (Figs. 2, 3). As in the case of cellulases, endoxylanases from the GH10 and GH11 families have been previously described to act synergistically, cleaving different substituted or unsubstituted regions in the polymer chain [38].

Most of the hydrolytic enzymes expressed by O199 showed identifiable signal peptides and were secreted extracellularly to the media. Extracellular cellulases and hemicellulases are notable from an industrial perspective because they considerably reduce the costs of the extraction procedures [3]. Moreover, many of secreted enzymes showed that CBM domains involved in cellulose and hemicellulose binding (Figs. 2, 3), which allow a strong interaction between the free enzymes and the substrates for efficient hydrolysis of cellulose and hemicellulose. Additionally, four proteins—an endoxylanase (ID161), a licheninase (ID3888), an α-galactosidase (ID5332) and a cellulose-binding protein (ID1552)—contained SLH domains, which mediate the binding of the protein to the cell surface [2]. SLH domains in hydrolytic enzymes contribute to efficient plant polysaccharide degradation, binding the enzymes to the cell surface and allowing the oligomers released in the proximity of the membrane to be immediately transported into the cell [39]. The fact that the SLH-containing proteins also contain CBMs binding cellulose (ID1552) or hemicelluloses (ID161, ID3888, ID5332) indicates that O199 cells are associated with lignocellulose, which highly increases the efficiency of decomposition. In this way, similar proteins containing SLH domains have been also described in other *Paenibacillus* strains. For example, a multimodular protein containing SLH domains and five CBMs—homologue of ID3888—have been recently described to be also involved in binding glucans through the CBM domains and in sequestering the polysaccharides to the cell surface for allowing the rapid transport of oligosaccharides released into the cell [40]. An homologue of ID161—a GH10 xylanase containing SLH and CBM9 and CBM22 domains—has been also defined as essential for xylan utilization in one strain of *Paenibacillus* and was also primarily involved in the generation of oligomers acting as inducers for the rest of the xylanase genes [41].

Despite the fact that some cellulases and hemicellulases were only expressed on one of the tested carbon sources, most of the hydrolytic enzymes were found on both substrates (Figs. 2, 3). In *Paenibacillus*, it has been found that both xylanases and cellulases were induced by cellulose, xylan, or complex lignocellulosic substances [42]. In this study, crystalline cellulose was able to induce hemicellulolytic enzymes even when hemicellulose was not present in the media. Because cellulose is always closely associated with other polymers, such as hemicelluloses and pectin, in plant material, the sole presence of cellulose or cellodextrins in the media may theoretically act as a general inducer of the whole enzymatic system for plant biomass degradation. This general induction may be more efficient than complex regulatory systems, allowing the bacteria to use a wide variety of plant polymers present in the soil environment. However, the presence of numerous transcriptional regulators, two-component regulatory systems, and sigma factors surrounding the genes coding for CAZymes—also reported in other *Firmicutes* cellulolytic strains [43, 44]—suggests that lignocellulose degradation by O199 is likely under complex regulation. Although it is known that oligomers from cellulose and hemicellulose degradation can act as inducers of cytoplasmic or membrane-associated accessory enzymes [4], the regulatory systems that respond to the presence of cellulose degradation products remain unknown in most bacteria [45]. In the cellulolytic fungi *Trichoderma reseei*, the presence of ABC transporters in cell membranes has been related to the induction of cellulases [10]. ABC transporters were abundantly found in O199 and in the genomes of other *Paenibacillus* species [31]. Their proximity to genes encoding CAZymes has also been suggested as an indicator of their involvement in the transport of sugars released by hydrolytic enzymes [33]. In bacteria, Xu et al. [46] showed that soluble saccharides are captured by SBPs from ABC transporters in *C. cellulolyticum*, inducing the activation of more transporters and CAZyme genes. In this study, at least forty-two putative SBPs involved in the binding of sugars were expressed during growth on straw and cellulose. Recent studies in other cellulolytic bacteria, such as *Caldicellulosiruptor bescii*, have reported that these secreted noncatalytic proteins are capable of binding a variety of plant cell wall soluble and insoluble saccharides, including microcrystalline cellulose, amorphous cellulose, xyloglucan, xylan and mannan, among others [47]. Therefore, the high amount of SBPs found in the proteomes of O199 may also explain the high efficiency shown by this isolate in filter paper degradation because substrate binding is an important prerequisite for the degradation of insoluble polysaccharides. However, unlike the CBMs, the role of these proteins secreted by cellulolytic bacteria is still poorly understood [47]. The fact that a high percentage of the proteins expressed on plant biomass and on cellulose were annotated as hypothetical or showed no functional prediction (Figure S3B and S3C) reflects the present lack of understanding.

## Conclusions

Methods for developing enzymatic cocktails for more efficient conversion of plant biomass into “green” energy are mainly based on improving the knowledge of all the players taking part in this process and in understanding the characteristics, dynamics and synergies between these enzymes and other involved proteins. The search for new cellulolytic isolates and analysis of their hydrolytic arsenal through the study of their genomes and proteomes are revealed as a promising strategy for ultimately enhancing the biomass conversion process. Here, we show that a newly described cellulolytic strain of *Paenibacillus* produced a rich and complex set of proteins for the complete deconstruction of cellulose and hemicellulose. Its high efficiency for cellulose hydrolysis is due to multiple and diverse enzymes showing different specificities, together with the presence of carbohydrate-binding domains and other nutrient-binding proteins, through the synergistic action of all of them. Interestingly, we found that most of the cellulolytic and hemicellulolytic enzymes were induced not only by complex plant biomass but also by cellulose.

The results support the use of the strain *Paenibacillus* O199 as a candidate for second-generation technologies using paper or lignocellulosic agricultural wastes (like wheat straw) as an inexpensive and sustainable alternative for the production of value-added chemicals and biofuels.

## Methods

### Strain isolation, identification and enzymatic activity

*Paenibacillus* O199 was isolated from the forest floor of a temperate sessile oak forest (*Quercus petraea*) in the Czech Republic. Litter chemistry and decomposition processes were studied in the selected area previously [48, 49], as well as the composition of bacterial communities in the litter and soil [50]. Strain isolation was performed by plating the forest floor material extracted with Ringer solution (100 mL g^−1^) on CMC agar medium (2 g L^−1^ yeast extract, 5 g L^−1^ carboxymethylcellulose (CMC), 50 mg L^−1^ of cycloheximide, pH 7.0) and incubated at 25 °C. After 7 days, agar plates were stained with 0.1 % Congo Red, and cellulose-degrading bacterial colonies showed clear halos indicating CMC degradation.

The ability of bacterial isolates to decompose cellulose was tested during growth in minimal medium with cellulose (filter paper strips of 20 mg weight) as the sole carbon source. The isolate O199 exhibited the fastest cellulose decomposition among all isolated strains. Bacteria were grown for 7 days at 25 °C on an orbital shaker. After that time, the integrity of the filter paper in the media was observed, and the activity of enzymes involved in the degradation of plant polymers was measured according to Valášková et al. [51]. Briefly, the activities of cellobiohydrolase (exocellulase), β-glucosidase, β-xylosidase, α-arabinosidase, glucuronidase, β-galactosidase, α-glucosidase and β-mannosidase were measured using methylumbelliferyl (MUF)-based substrates on a microplate reader (Infinite, TECAN, Groedig, Austria), with an excitation wavelength of 355 nm and an emission wavelength of 460 nm. Calibration of product development was based on standard curves with a range of MUF concentrations added to the sample.

The bacterial 16S rRNA gene of the strain O199 was sequenced using the primers 27F and 1492R [52]. The EzTaxon server (http://www.ezbiocloud.net/eztaxon) [53] was used for isolate identification, using the EzTaxon database that contains 16S rDNA sequences of the type strains of species. The sequence of the 16S ribosomal RNA gene was deposited in the GenBank database under the accession number KR181834.

### DNA extraction, whole-genome sequencing and genome analysis

Total genomic DNA was extracted from bacteria grown in GYM media (4 g L^−1^ glucose; 4 g L^−1^ yeast extract; 10 g L^−1^ malt extract; pH 7.0) with the UltraClean Microbial DNA Isolation Kit (MoBio Laboratories, Carlsbad, CA, USA), and sequencing was performed on the Illumina MiSeq platform in a paired-end 2 × 250 bp run. The sequence data were assembled using Velvet 1.2.10 [54], and a draft genome was obtained. Draft genome sequences were deposited in GenBank under the accession number LGRP00000000. Gene annotation was performed using Rapid Annotations Subsystems Technology (RAST) 4.0 [55, 56]. To identify the CAZymes, translated proteins from the predicted open reading frames were analysed with dbCAN [57]. Information about the carbohydrate-active enzyme content in the complete genomes of closely related bacteria was obtained from the CAZy database [6].

### Protein expression and GeLC-MS/MS analysis of extracellular proteins

For the analysis of protein expression, bacteria were pre-grown in GYM media for 24 h, and 1 mL of culture was inoculated in triplicate in 1 L of MM containing 0.5 % w/v of either microcrystalline cellulose (Serva, Heidelberg, Germany) or wheat straw that was finely milled and repeatedly washed with hot water to remove low-molecular mass compounds while retaining plant cell wall biopolymers. Cultures were incubated for 7 days at 25 °C in an orbital shaker. After harvest, cultures were centrifuged, and proteins in the supernatant were precipitated with 10 % w/v trichloroacetic acid and resuspended in 8 M urea/2 M thiourea buffer. Protein concentration was measured in every sample with Roti^®^-Nanoquant (Carl Roth, GmbH, Germany), and 25 μg was separated by 1D-SDS-PAGE using Criterion™ TGX™ Precast Gels (Bio-Rad Laboratories, Hercules, CA, USA). Lanes were cut in ten equidistant pieces and subjected to trypsin digestion as previously described [58]. Peptide mixtures were separated by RP chromatography using a nanoACQUITY™ UPLC™ system (Waters, Milford, MA, USA). Peptides were loaded onto a trap column and separated on the analytical column using a binary 80 min gradient of buffer B (99.9 % ACN, 0.1 % acetic acid) at a constant flow rate of 400 nL min^−1^. The UPLC system was coupled to an LTQ Orbitrap mass spectrometer (Thermo Fisher Scientific, Waltham, MA, USA). Full survey scans were recorded in the Orbitrap (*m*/*z* range from 300 to 2000) with a resolution of 30,000 and lock mass option enabled. MS/MS experiments in the LTQ XL were performed for the five most abundant precursor ions (CID), excluding unassigned charge states and singly charged ions. Dynamic exclusion was enabled after 30 s. For protein identification, spectra were searched against a database of *Paenibacillus* O199 containing sequences of all predicted proteins from its genome, including reverse sequences and sequences of common laboratory contaminants (13,098 entries). Database searches were performed using Sorcerer SEQUEST (Version v. 27 rev. 11, Thermo Scientific) and Scaffold 4.0.5 (Proteome Software, Portland, OR, USA) with the following search parameters: parent ion tolerance: 10 ppm, fragment ion mass tolerance: 1.00 Da, up to two missed cleavages were allowed and methionine oxidation (+15.99492 Da) was set as variable modification. For protein identification, a stringent SEQUEST filter for peptides was used (Xcorr versus charge state: 2.2, 3.3 and 3.8 for doubly, triply and quadruply charged peptides, respectively, and deltaCn value greater than 0.10), and at least two peptides per protein were required. Protein quantification was based on the normalized spectrum abundance factor (NSAF), which is calculated as the number of spectral counts (SpC) identifying a protein, divided by protein length (L), divided by the sum of SpC/L for all proteins in the experiment [59]. Statistical analysis was performed using MeV v4.8.1 [60]. Student’s *t* test was performed with the following settings: unequal group variances were assumed (Welch approximation), *P* values based on all permutations with *P* = 0.01, significance determined by adjusted Bonferroni correction. Functional prediction and classification of proteins were performed by the in-house developed analysis pipeline *Prophane 2.0* (http://www.prophane.de) [61] and the RAST annotation server. Voronoi treemaps were generated using Paver (Decodon, Greifswald, Germany; http://www.decodon.com/). Sequences of the identified proteins and those previously predicted to be GHs with dbCAN were compared with the characterized sequences deposited in the CAZy database for the GH families found in the proteome for functional prediction. For this purpose, sequences were aligned with MUSCLE 3.7 [62], and trees based on the maximum likelihood were constructed with PhyML 3.0 [63]. Where possible, the putative role for the identified GHs was directly assigned based on the closest sequences in the family tree whose functional roles were known. Identity between similar proteins was calculated using the Basic Local Alignment Search Tool for proteins (BLASTP, http://www.ncbi.nlm.nih.gov/). The mass spectrometry proteomics data have been deposited to the ProteomeXchange Consortium via the PRIDE [64] partner repository with the dataset identifier PXD003970.

## Additional files

10.1186/s13068-016-0518-x Cellulolytic ability of *Paenibacillus* O199. A) Growth in minimal medium containing filter paper as the sole carbon source. The deconstruction of the paper by bacterial enzymes was complete after 7 days (on the right), in comparison with the control (on the left). B) Activity of enzymes involved in the degradation of polysaccharides in the culture. “–“: no activity detected.
10.1186/s13068-016-0518-x Phylogenetic tree of *Paenibacillus* species based on complete 16S rRNA gene sequences. Strains described as cellulolytic (*) and hemicellulolytic (^x^) are marked. The tree was constructed via the maximum likelihood method (ML) using a Kimura 2-parameter model (K2) and a discrete gamma distribution with invariant sites (G + I) (bootstrap confidence levels determined by 500 bootstrap replications are shown as percentages of nodes) with the software package MEGA 5.1. Sequences were obtained from EzTaxon.
10.1186/s13068-016-0518-x Genome content and protein expression on wheat straw and on crystalline cellulose of carbohydrate-active enzymes of *Paenibacillus* O199.
10.1186/s13068-016-0518-x Proteomic analysis of *Paenibacillus* O199.
10.1186/s13068-016-0518-x Identification of proteins in the proteomes of *Paenibacillus* O199. A) Venn diagrams depicting identified proteins during growth on cellulose (red) and wheat straw (blue). B) Voronoi treemap visualization of protein expression pattern of *Paenibacillus* O199 during growth on cellulose (red) and wheat straw (blue). Functional classification of proteins was carried out by *Prophane 2.0* and RAST and is based on TIGRFAM classification. Each cell represents a protein; proteins are clustered according to their function. Functional classes are separated by thicker black lines.
10.1186/s13068-016-0518-x Summary of proteins annotated as substrate-binding proteins (SBP) from ATP-binding cassette (ABC) transporters detected in the proteomes of *Paenibacillus* O199. Annotation was performed with RAST.

## Abbreviations

CAZyme: carbohydrate-active enzyme
GH: glycosyl hydrolase
CBM: carbon-binding domain
CE: carbohydrate esterase
PL: polysaccharide lyase
AA: auxiliary activities
LPMO: lytic polysaccharide monooxygenase
CMC: carboxymethylcellulose
ABC: ATP-binding cassette
PTS: phosphotransferase system
SLH: surface-layer homology
SBP: substrate-binding protein
NSAF: normalized spectrum abundance factor
SpC: spectral counts
GeLC-MS/MS: one-dimensional polyacrylamide gel electrophoresis liquid chromatography tandem mass spectrometry
