# Supplementary material for: Decoding the complete arsenal for cellulose and hemicellulose deconstruction in the highly efficient cellulose decomposer Paenibacillus O199
Source: Biotechnol Biofuels. 2016 May 14;9:104. doi: 10.1186/s13068-016-0518-x (PMC4867992; doi:10.1186/s13068-016-0518-x)
Supplement: Supplementary file 1 — 10.1186/s13068-016-0518-x Cellulolytic ability of Paenibacillus O199. A) Growth in minimal medium containing filter paper as the sole carbon source. The deconstruction of the paper by bacterial enzymes was complete after 7 days (on the right), in comparison with the control (on the left). B) Activity of enzymes involved in the degradation of polysaccharides in the culture. “–“: no activity detected. [file 13068_2016_518_MOESM1_ESM.pdf]

Figure S1. Cellulolytic ability of *Paenibacillus* O199. A) Growth in minimal medium containing filter paper as the sole carbon source. The deconstruction of the paper by bacterial enzymes was complete after 7 days (on the right), in comparison with the control (on the left). B) Activity of enzymes involved in the degradation of polysaccharides in the culture. “–“: no activity detected.

A

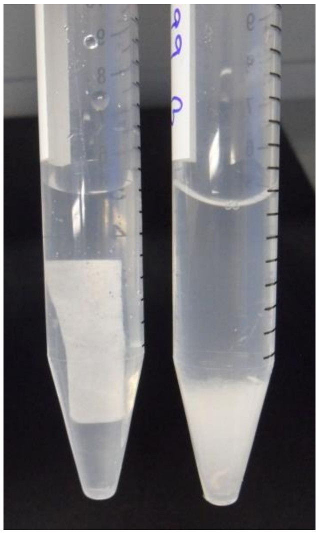

B

**Enzymatic activity**	mU/mL
Cellobiohydrolase	27
β-glucosidase	149
β-xylosidase	10
α-arabinosidase	47
Glucuronidase	-
β-galactosidase	330
α-glucosidase	-
β-mannosidase	114
