# Supplementary material for: Decoding the complete arsenal for cellulose and hemicellulose deconstruction in the highly efficient cellulose decomposer Paenibacillus O199
Source: Biotechnol Biofuels. 2016 May 14;9:104. doi: 10.1186/s13068-016-0518-x (PMC4867992; doi:10.1186/s13068-016-0518-x)

Figure S2. Phylogenetic tree of *Paenibacillus* species based on complete 16S rRNA gene sequences. Strains described as cellulolytic (*) and hemicellulolytic (ˣ) are marked. The tree was constructed via the maximum likelihood method (ML) using a Kimura 2-parameter model (K2) and a discrete gamma distribution with invariant sites (G+I) (bootstrap confidence levels determined by 500 bootstrap replications are shown as percentages of nodes) with the software package MEGA 5.1. Sequences were obtained from EzTaxon.

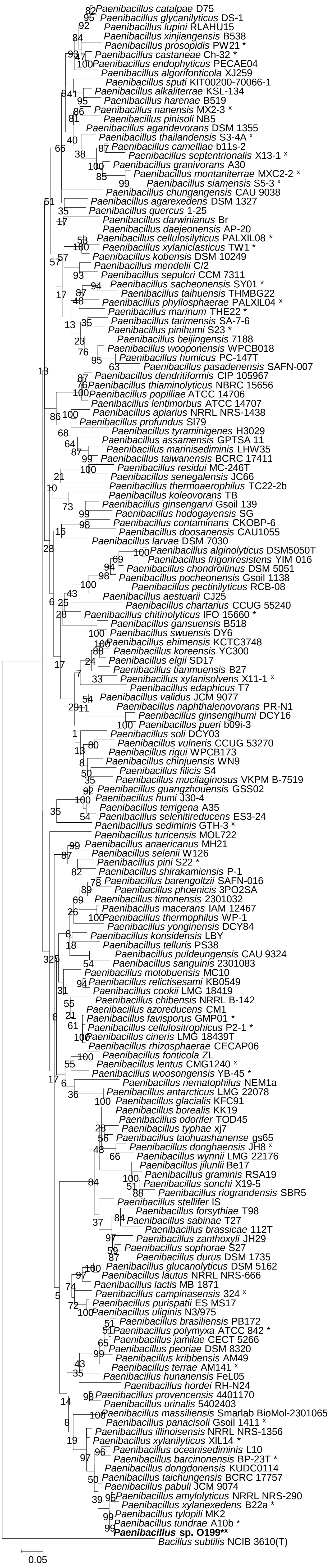

Supplement: Supplementary file 2 — 10.1186/s13068-016-0518-x Phylogenetic tree of Paenibacillus species based on complete 16S rRNA gene sequences. Strains described as cellulolytic (*) and hemicellulolytic (x) are marked. The tree was constructed via the maximum likelihood method (ML) using a Kimura 2-parameter model (K2) and a discrete gamma distribution with invariant sites (G + I) (bootstrap confidence levels determined by 500 bootstrap replications are shown as percentages of nodes) with the software package MEGA 5.1. Sequences were obtained from EzTaxon. [file 13068_2016_518_MOESM2_ESM.pdf]
