# Supplementary material for: Decoding the complete arsenal for cellulose and hemicellulose deconstruction in the highly efficient cellulose decomposer Paenibacillus O199
Source: Biotechnol Biofuels. 2016 May 14;9:104. doi: 10.1186/s13068-016-0518-x (PMC4867992; doi:10.1186/s13068-016-0518-x)
Supplement: Supplementary file 3 — 10.1186/s13068-016-0518-x Genome content and protein expression on wheat straw and on crystalline cellulose of carbohydrate-active enzymes of Paenibacillus O199. [file 13068_2016_518_MOESM3_ESM.docx]

Table S1. Genome content and protein expression on wheat straw and on crystalline cellulose of carbohydrate-active enzymes of *Paenibacillus* O199.

| CAZy family | **Genome** | **Wheat straw** | **Cellulose** |
| --- | --- | --- | --- |
| GH1 | 11 | 1 | 1 |
| GH2 | 10 | 2 | 3 |
| GH3 | 11 | 2 | 4 |
| GH4 | 6 | 0 | 0 |
| GH5 | 7 | 3 | 3 |
| GH6 | 1 | 1 | 1 |
| GH9 | 1 | 0 | 1 |
| GH10 | 3 | 3 | 2 |
| GH11 | 1 | 1 | 0 |
| GH12 | 1 | 1 | 1 |
| GH13 | 12 | 2 | 4 |
| GH16 | 5 | 4 | 4 |
| GH18 | 8 | 3 | 5 |
| GH20 | 1 | 0 | 0 |
| GH23 | 3 | 0 | 0 |
| GH25 | 1 | 1 | 1 |
| GH26 | 2 | 1 | 1 |
| GH27 | 1 | 0 | 0 |
| GH28 | 4 | 1 | 1 |
| GH29 | 6 | 0 | 1 |
| GH30 | 6 | 5 | 5 |
| GH31 | 2 | 1 | 1 |
| GH32 | 2 | 0 | 0 |
| GH33 | 1 | 0 | 0 |
| GH35 | 1 | 0 | 0 |
| GH36 | 4 | 2 | 2 |
| GH38 | 8 | 1 | 1 |
| GH42 | 4 | 1 | 1 |
| GH43 | 17 | 6 | 5 |
| GH46 | 1 | 0 | 0 |
| GH48 | 1 | 1 | 1 |
| GH51 | 4 | 2 | 2 |
| GH52 | 1 | 1 | 1 |
| GH53 | 3 | 2 | 2 |
| GH63 | 1 | 0 | 0 |
| GH65 | 1 | 0 | 0 |
| GH67 | 1 | 0 | 0 |
| GH73 | 1 | 0 | 0 |
| GH74 | 4 | 2 | 3 |
| GH76 | 2 | 1 | 1 |
| GH78 | 4 | 1 | 1 |
| GH79 | 0 | 0 | 0 |
| GH85 | 2 | 1 | 1 |
| GH87 | 1 | 1 | 1 |
| GH88 | 3 | 0 | 0 |
| GH94 | 2 | 0 | 0 |
| GH95 | 4 | 1 | 1 |
| GH99 | 2 | 0 | 1 |
| GH101 | 1 | 0 | 0 |
| GH105 | 5 | 1 | 1 |
| GH106 | 3 | 0 | 0 |
| GH109 | 28 | 10 | 10 |
| GH110 | 1 | 0 | 0 |
| GH112 | 2 | 0 | 0 |
| GH113 | 1 | 0 | 0 |
| GH123 | 1 | 0 | 0 |
| GH124 | 1 | 0 | 0 |
| GH125 | 3 | 0 | 0 |
| GH126 | 1 | 0 | 0 |
| GH127 | 1 | 0 | 0 |
| GH129 | 2 | 0 | 1 |
| GH130 | 3 | 0 | 1 |
| CBM3 | 5 | 4 | 5 |
| CBM4 | 2 | 1 | 0 |
| CBM6 | 5 | 1 | 4 |
| CBM9 | 4 | 1 | 2 |
| CBM12 | 3 | 3 | 3 |
| CBM13 | 4 | 4 | 4 |
| CBM20 | 1 | 1 | 1 |
| CBM22 | 1 | 1 | 1 |
| CBM32 | 12 | 6 | 7 |
| CBM34 | 3 | 0 | 0 |
| CBM35 | 6 | 4 | 5 |
| CBM37 | 3 | 0 | 1 |
| CBM38 | 2 | 0 | 0 |
| CBM40 | 1 | 0 | 0 |
| CBM41 | 1 | 0 | 1 |
| CBM42 | 2 | 1 | 1 |
| CBM46 | 5 | 3 | 3 |
| CBM48 | 3 | 0 | 1 |
| CBM50 | 11 | 2 | 2 |
| CBM51 | 2 | 0 | 0 |
| CBM54 | 1 | 0 | 1 |
| CBM56 | 1 | 1 | 1 |
| CBM61 | 1 | 0 | 1 |
| CBM66 | 2 | 1 | 1 |
| CBM67 | 3 | 1 | 1 |
| AA6 | 7 | 1 | 1 |
| AA7 | 2 | 0 | 0 |
| AA10 | 1 | 1 | 1 |
| CE1 | 27 | 5 | 5 |
| CE2 | 2 | 0 | 1 |
| CE3 | 5 | 5 | 3 |
| CE4 | 12 | 1 | 0 |
| CE7 | 4 | 1 | 1 |
| CE8 | 1 | 0 | 0 |
| CE9 | 4 | 1 | 1 |
| CE10 | 17 | 3 | 3 |
| CE11 | 1 | 0 | 0 |
| CE12 | 3 | 2 | 1 |
| CE14 | 2 | 1 | 1 |
| CE15 | 1 | 1 | 1 |
| PL1 | 3 | 2 | 2 |
| PL3 | 1 | 1 | 1 |
| PL4 | 1 | 1 | 1 |
| PL9 | 3 | 2 | 1 |
| PL10 | 1 | 0 | 0 |
| PL11 | 3 | 3 | 1 |
| PL12 | 1 | 0 | 0 |
| PL15 | 1 | 0 | 0 |
| PL22 | 1 | 0 | 0 |
| GT1 | 2 | 0 | 0 |
| GT2 | 24 | 0 | 0 |
| GT4 | 15 | 0 | 0 |
| GT5 | 1 | 0 | 0 |
| GT19 | 1 | 0 | 0 |
| GT26 | 3 | 0 | 0 |
| GT27 | 1 | 0 | 0 |
| GT28 | 5 | 0 | 0 |
| GT35 | 1 | 0 | 0 |
| GT46 | 1 | 0 | 0 |
| GT51 | 4 | 0 | 1 |
| GT62 | 2 | 0 | 0 |
| GT83 | 1 | 0 | 0 |
