# Supplementary material for: Decoding the complete arsenal for cellulose and hemicellulose deconstruction in the highly efficient cellulose decomposer Paenibacillus O199
Source: Biotechnol Biofuels. 2016 May 14;9:104. doi: 10.1186/s13068-016-0518-x (PMC4867992; doi:10.1186/s13068-016-0518-x)
Supplement: Supplementary file 6 — 10.1186/s13068-016-0518-x Summary of proteins annotated as substrate-binding proteins (SBP) from ATP-binding cassette (ABC) transporters detected in the proteomes of Paenibacillus O199. Annotation was performed with RAST. [file 13068_2016_518_MOESM6_ESM.docx]

Table S2. Summary of proteins annotated as substrate-binding proteins (SBP) from ATP-binding cassette (ABC) transporters detected in the proteomes of *Paenibacillus* O199. Annotation was performed with RAST.

| Substrate | Description | Protein ID | Total |
| --- | --- | --- | --- |
| Undefined | ABC transporter, substrate-binding protein | 1585, 1595, 1600, 2267, 2451, 2782, 2877, 3123, 3204, 3568, 3753, 3798, 3829, 3972, 4627, 4742, 5036, 5313, 5338, 5673, 5901, 5968, 6135, 6191, 6253, 6452 | 26 |
| Sugars | Sugar ABC transporter substrate-binding protein | 999, 3174, 3815, 3882, 4703, 4772, 5286, 5560 | 8 |
|  | Multiple sugar ABC transporter, substrate-binding protein | 2359, 3864, 4948, 5145, 5290, 5702, 5811, 5985 | 8 |
|  | Polysaccharide ABC transporter substrate-binding protein | 2265, 4318, 4319, 5326 | 4 |
|  | Xylose ABC transporter, substrate-binding component | 3355 | 1 |
|  | N-Acetyl-D-glucosamine ABC transport system, sugar-binding protein | 377, 700, 981, 1264, 1556, 1649, 1984, 2772, 3737, 3856, 4278, 4329, 4890, 5871, 6026, 6094, 6453 | 17 |
|  | Predicted rhamnose oligosaccharide ABC transport system, substrate-binding component | 3712 | 1 |
|  | Lactose transport system (lactose-binding protein) | 5228 | 1 |
|  | Maltose/maltodextrin ABC transporter, substrate binding periplasmic protein MalE | 1323, 2842 | 2 |
| Peptides and aminoacids | Oligopeptide ABC transporter, periplasmic oligopeptide-binding protein OppA (TC 3.A.1.5.1) | 1093, 1174, 1096, 1422, 1753, 2031, 2458, 2537 | 8 |
|  | Dipeptide-binding ABC transporter, periplasmic substrate-binding component (TC 3.A.1.5.2) | 739, 2349, 2707, 2941 | 4 |
|  | Glutamine ABC transporter, periplasmic glutamine-binding protein (TC 3.A.1.3.2) | 6446 | 1 |
|  | Methionine ABC transporter substrate-binding protein | 5731 | 1 |
| Others | Predicted nucleoside ABC transporter, substrate-binding component | 4956, 6428 | 2 |
|  | Glycerol-3-phosphate ABC transporter, periplasmic glycerol-3-phosphate-binding protein (TC 3.A.1.1.3) | 4746 | 1 |
|  | Vitamin B12 ABC transporter, B12-binding component BtuF | 2515, 3108 | 2 |
|  | Inositol transport system sugar-binding protein | 2922 | 1 |
|  | Lipoprotein | 565, 567, 2262, 3000, 3195 | 5 |
|  | ABC-type Fe3+-siderophore transport system, periplasmic iron-binding component | 2002 | 1 |
|  | Manganese ABC transporter, periplasmic-binding protein SitA | 5744 | 1 |
