# Supplementary material for: Decoding the complete arsenal for cellulose and hemicellulose deconstruction in the highly efficient cellulose decomposer Paenibacillus O199
Source: Biotechnol Biofuels. 2016 May 14;9:104. doi: 10.1186/s13068-016-0518-x (PMC4867992; doi:10.1186/s13068-016-0518-x)

Figure S3. Identification of proteins in the proteomes of *Paenibacillus* O199. A) Venn diagrams depicting identified proteins during growth on cellulose (red) and wheat straw (blue). B) and C) Voronoi treemap visualization of expressed protein: functional classification of common proteins in both carbon sources (B); expression pattern of proteins during growth on cellulose (red) and wheat straw (blue) (C). Functional classification of proteins was carried out by *Prophane* 2.0 and RAST and is based on TIGRFAM classification. Each cell represents a protein; proteins are clustered according to their function. Functional classes are separated by thicker black lines.

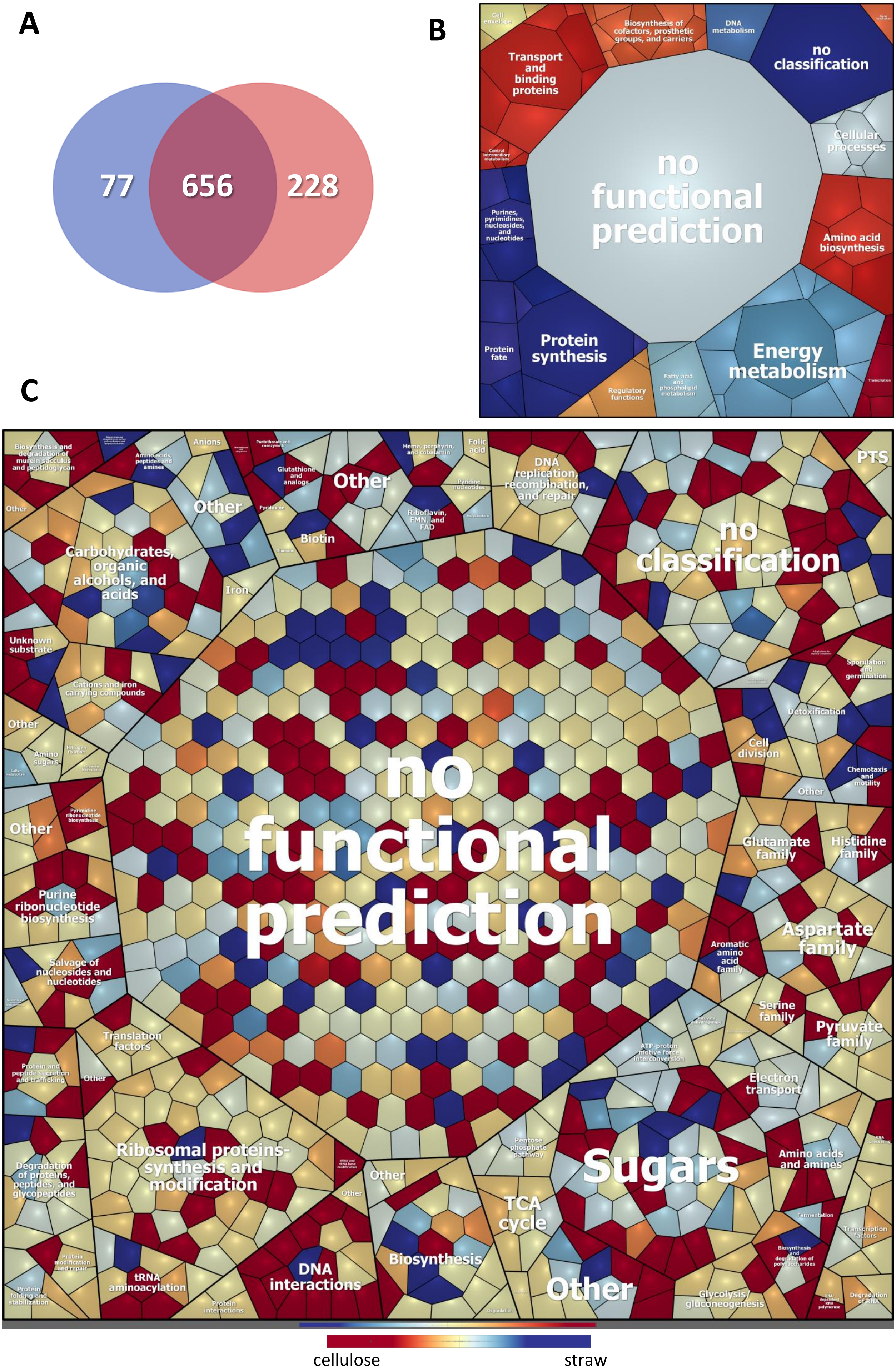

Supplement: Supplementary file 5 — 10.1186/s13068-016-0518-x Identification of proteins in the proteomes of Paenibacillus O199. A) Venn diagrams depicting identified proteins during growth on cellulose (red) and wheat straw (blue). B) Voronoi treemap visualization of protein expression pattern of Paenibacillus O199 during growth on cellulose (red) and wheat straw (blue). Functional classification of proteins was carried out by Prophane 2.0 and RAST and is based on TIGRFAM classification. Each cell represents a protein; proteins are clustered according to their function. Functional classes are separated by thicker black lines. [file 13068_2016_518_MOESM5_ESM.pdf]
